# The Effect of Broccoli Extract in Arsenic-Induced Experimental Poisoning on the Hematological, Biochemical, and Electrophoretic Parameters of the Liver and Kidney of Rats

**DOI:** 10.1155/2022/3509706

**Published:** 2022-01-05

**Authors:** Mahdieh Raeeszadeh, Pouria Karimi, Nadia Khademi, Pejman Mortazavi

**Affiliations:** ^1^Department of Basic Sciences, Islamic Azad University, Sanandaj Branch, Sanandaj, Iran; ^2^Graduate of Faculty of Veterinary Sciences, Islamic Azad University, Sanandaj Branch, Sanandaj, Iran; ^3^Department of Pathobiology Sciences, Islamic Azad University, Science and Research Branch, Tehran, Iran

## Abstract

Heavy metals such as arsenic contribute to environmental pollution that can lead to systemic effects in various body organs. Some medicinal plants such as broccoli have been shown to reduce the harmful effects of these heavy metals. The main aim of the present study is to evaluate the effects of broccoli extract on liver and kidney toxicity, considering hematological and biochemical changes. The experimental study was performed in 28 days on 32 male Wistar rats classified into four groups: the control group (C), a group receiving 5 mg/kg oral arsenic (AS), a group receiving 300 mg/kg broccoli (B), and a group receiving arsenic and broccoli combination (AS + B). Finally, blood samples were taken to evaluate the hematological and biochemical parameters of the liver and kidney, as well as serum proteins' concentration. Liver and kidney tissue were fixed and stained by H&E and used for histopathological diagnosis. The results demonstrated a significant decrease in white blood cells (WBC), red blood cells (RBC), and hemoglobin (Hb) in the AS group compared to other groups. However, in the B group, a significant increase in RBC and WBC was observed compared to the AS and C groups (*P* < 0.05). Moreover, RBC and WBC levels increased significantly in the AS + B group compared to the AS group (*P* = 0.046). However, in the AS group, aspartate aminotransferase (AST), alanine aminotransferase (ALT), urea, and creatinine levels increased, while total protein, albumin, and globulin decreased. This can be a result of liver and kidney damage, which was observed in the AS group. Furthermore, the increase in the concentration of albumin and globulin in the AS + B group was higher than that in the AS group. Infiltration of inflammatory cells and necrosis of the liver and kidney tissue in the pathological evaluation of the AS group were significantly higher than other groups. There was an increase in superoxide dismutases (SOD), glutathione peroxidase (GPx), and total antioxidant capacity (TAC); however, a decrease in malondialdehyde (MDA) concentration was seen in the AS + B group compared to the AS group. It seems that broccoli is highly effective at reducing liver and kidney damage and improving the hematological and biochemical factors in arsenic poisoning conditions.

## 1. Introduction

Arsenic ranks second (after lead) among the significant heavy metals, while arsenic contamination is one of the major global environmental problems [1]. This poisonous substance endangers humans' and animals' life. Because of the extensive human industrial activities and the abundance of chemical pollutants, pesticides, and herbicides, this heavy metal in the ecosystem is increasing progressively [2]. The most common cause of arsenic contamination is eating and drinking, followed by inhalation and dermal absorption [3]. Arsenic poisoning changes the hematological, biochemical, and oxidative stress parameters and leads to tissue damage (mainly in the liver and kidney). This is supported by evidence from carcinogenicity in animals and humans [4].

Various mechanisms have been reported for arsenic poisoning, including induction of oxidative stress, inhibition of enzymes, and changes in the mitochondrial function. Moreover, binding with sulfhydryl (-SH) groups is another pathway for arsenic damage [5].

Experimental studies on the liver (being the most vulnerable organ to arsenic) have reported that arsenic poisoning can change the concentration of liver enzymes. Liver damage is mainly related to ROS production and oxidative stress [6]. Chronic arsenic poisoning also increases the acute-phase protein and C-reactive protein (CRP) in the liver and kidney and raises proinflammatory cytokines [7].

Oxidative stress and inflammation increase transcription factor activation and the gene expression of proinflammatory cytokines as a complex cycle [8]. Decreased phagocytic activity in leukocytes under the influence of oxidative damage can further amplify inflammatory reactions [9].

Recently, researchers have focused on the safe and available treatment methods and prevention of heavy metal poisoning. The use of chelating agents such as dimercaptopropane and binding to antioxidant compounds such as vitamins C and E have been suggested [10, 11]. On the other hand, increasing the concentration of intracellular antioxidants can also be helpful in preventing arsenic poisoning. Protection against calcium homeostasis and mitochondrial integrity that regulates apoptosis is another effective mechanism that has a positive impact on arsenic poisoning [12].

Vegetables as a source of antioxidants with almost no side effects for physiological systems are essential for human health [13]. Broccoli (*Brassica oleracea* L. var. *Italica*) contains a variety of polyphenols, so it greatly benefits the body. The natural antioxidants in broccoli include beta-carotene, vitamins C and E, which may directly and/or indirectly help reduce free radicals. Other antioxidants of broccoli such as iron, selenium, calcium, manganese, phosphorus, zinc, and potassium help regulate the body's ionic balance [14].

Considering the importance of controlling and preventing arsenic poisoning and the lack of research emphasis in this area, the aim of this study was to investigate the effect of broccoli extract on changes caused by arsenic poisoning in blood and the biochemical parameters in the liver and kidney of rats.

## 2. Materials and Methods

### 2.1. Subjects

The study was performed on 32 male Wistar rats weighing 200–230 g. The animals were housed in standard conditions, with temperatures between 20 and 22°C, humidity ranging from 45 to 50%, 12 hours of light, and 12 hours of darkness in the animal house of Sanandaj Veterinary Medicine university. The study followed the rules and regulations of animal ethics and the approval of the Ethics Committee of Kurdistan Medical Sciences (IR.MUK.REC.1400.6089).

### 2.2. Experimental Protocol

The animals were randomly divided into four groups (8 in each group): the control group (C), receiving no treatment; the arsenic group (AS), receiving sodium arsenate, CAS Number 10048-95-0, at 5 mg/kg/body weight by means of oral gavage; the broccoli group (B), receiving broccoli extract at a dose of 300 mg/kg (based on the best functional dose in previous studies) intraperitoneal (IP) injection; and the AS + B group (AS + B), receiving 5 mg/kg sodium arsenate and 300 mg/kg broccoli alcoholic extract [15, 16].

The study lasted 28 days, with blood samples taken from animals under anesthesia on the last day. EDTA-anticoagulant blood was used to measure hematological parameters such as red blood cells (RBC), white blood cells (WBC), hematocrit (HCT), neutrophils, lymphocytes, monocytes, eosinophils, and hemoglobin (HGB). Serum was separated by centrifugation. It was used to estimate hepatic and renal biochemical parameters including aspartate transaminase (AST), alanine aminotransferase (ALT), alkaline phosphatase (ALP), creatinine, and urea. Furthermore, oxidative stress levels glutathione peroxidase (GPx), superoxide dismutase (SOD), catalase (CAT), total antioxidant capacity (TAC), and malondialdehyde (MDA) were measured. Moreover, changes in serum proteins regarding albumin and globulin were evaluated by electrophoresis.

### 2.3. Hematological Parameters

Hemoglobin concentration was determined by the cyanmethemoglobin method. The RBC and WBC counts were measured using a Neubauer hemocytometer. The differential leukocyte counts were measured by exploiting standard methods [4].

### 2.4. Serum Biochemical Analysis

ALP, AST, ALT, urea, and creatinine levels were measured using a Pars test kit and an autoanalyzer [4].

### 2.5. Stress Oxidative Biomarkers

Trichloroacetic acid (TCA) was measured using the Ferric reducing antioxidant power (FRAP) method suggested by Benzi et al. [17].

Thiobarbituric acid (TBA) reactivity was measured using malondialdehyde (MDA). 1 ml of reagent [10% trichloroacetic acid (TCA), 0.67% thiobarbituric acid (TBA), and 0.25 M hydrochloric acid (HCl)] was added to 500 *µ*l of serum sample. This mixture was then placed in a bain-marie for 15 minutes at 95°C. After cooling, the mixture was centrifuged (10 min, 1000 g), and the obtained supernatant absorbance was determined at 535 nm [18].

The catalase enzyme decomposes hydrogen peroxide into water and oxygen, measured spectrophotometrically at a wavelength of 240 nm.

The superoxide dismutase enzyme was measured at 505 nm [19]. To this end, xanthine and xanthine oxidase (XOD) were used to produce superoxide radicals, in which the reaction with the combination phenyl tetrazolium chloride-5-(nitrophenol-4)-3-(iodophenol-4)-2 creates red dye color.

Glutathione peroxidase was measured using the method described by Paglia and Valentine. The glutathione peroxidase enzyme catalyzes the oxidation reaction of glutathione (GSH) via cumene hydroperoxide that can be measured by light absorption at a wavelength of 340 nm [20].

### 2.6. Serum Protein Electrophoresis (SPEP)

SPEP was measured by electrophoresis on cellulose acetate paper. The steps are as follows:

- Cellulose acetate paper was placed in barbital buffer for 10 minutes at pH = 8.6
- Cellulose acetate paper damping was done between two sheets of filter paper
- Ten microliters of serum from each sample was poured on each template platform
- Sampling was done 4–6 times by the applicator at a distance of 3 cm from the edge of the cellulose acetate paper
- The gel was placed on the tank and electrophoresis was performed for 30 minutes at 160 volts and a current between 5–7 mA
- At the end of the electrophoresis time, the plate(s) was (were) removed from the chamber. They were placed in 40–50 mL of Ponceau S Staining sufficient volume to cover the plate(s) for 5–7 minutes of shaking
- Destaining was done in three consecutive containers of 5% acetic acid, each for 2 to 3 minutes.
- Dehydration was done in pure methanol for 2 minutes
- The plate was placed in the clearing solution for 5–10 minutes
- The plate was placed in a drying oven at 70°C for 10 minutes or until dry
- The cleared film, when cooled, was ready for the densitometer scanner
- Charts were drawn and printed [21, 22]

### 2.7. Histopathological Evaluation of the Liver and Kidney

Liver and kidney tissue were fixed in 10% formalin buffer after separation. Then, tissue sections were prepared. Hematoxylin-eosin (H and E) staining was performed.

Liver and kidney tissue sections were analyzed based on the severity and weakness of tissue lesions at a magnification of 400 in 5 microscopic fields.

### 2.8. Preparation of Alcoholic Extract of Broccoli

Due to the higher antioxidant of alcoholic broccoli extract than aqueous extract, methanolic extract was considered. Therefore, after drying and grinding the broccoli, 50 g of powder was kept in 250 ml of 80% methanol by shaking for 48 hours. Then, after filtering the extract, it was placed in a rotary vacuum to let the dissolvent be evaporated. Then, the extract was dried into a powder and stored in refrigeration for administration at a dose of 300 mg/kg [16, 23, 24].

### 2.9. Data Analysis

After checking the normality of the data using the Kolmogorov–Smirnov test, the descriptive data were reported as mean ± standard deviation. After that, the results were analyzed using one-way analysis of variance (ANOVA), followed by Tukey post hoc test. The significance level was set at *P* < 0.05.

## 3. Results

According to Table 1, the hematological analysis showed that the highest mean of WBC is in the B group, and the lowest is in the AS group. The WBC in the B and AS + B groups was significantly different from other groups. The lowest mean of the RBC was shown in the AS group, and the highest mean was reported in the B group. Moreover, the increased hemoglobin concentration in the B group is significantly higher than in other groups except for the C group. On the other hand, the PCV percentage was not significantly different between groups. Additionally, the levels of neutrophils, lymphocytes, monocytes, and eosinophils did not show a significant difference between groups.

The biochemical changes in the liver and kidney parameters are shown in Table 2. The concentration of ALT, as a liver-specific enzyme, was 74.3 ± 1.90 in the AS group, which was the highest value reported between groups, but after treatment with broccoli, it reduced to 58.60 ± 6.60. The difference between the S group and other groups was significant. Serum AST and ALP levels were highest in the AS group. However, the enzyme values did not differ significantly between the B and C groups. The enzyme levels decreased in the AS + B group, although AST concentration in this group was not significantly different from the control group.

Creatinine concentration in AS-induced renal injury has the highest value among groups (1.42 ± 0.18) and is significantly different from other groups. Urea showed the lowest values in the C group and the highest values in the AS group. The serum urea concentration in the AS group was significantly dissimilar to other groups except for the AS + B group. There was also a significant difference in the serum urea level between the AS and B groups.

Table 3 shows the changes in oxidative stress biomarkers. The level of CAT enzyme was different between the B and AS groups, but the differences between the other groups were not significant. GPx enzyme showed the highest serum levels in the C and B groups, and the lowest value was found in the AS group. There was a statistically significant difference between the AS group and other experimental groups. Furthermore, SOD enzyme levels were highest in the C and B groups. However, the antioxidant enzyme levels increased after treatment with broccoli extract compared to the AS group. The highest concentration of MDA was observed in the AS group (12.45 ± 1.20 nmol/ml), which showed a significant difference with the other groups. Evaluation of TAC showed the highest values in the B group (858.47 ± 25.49) compared to the lowest values in the AS group (220.29 ± 69.53). These two groups were significantly different from other experimental groups.

**Figure 1:**
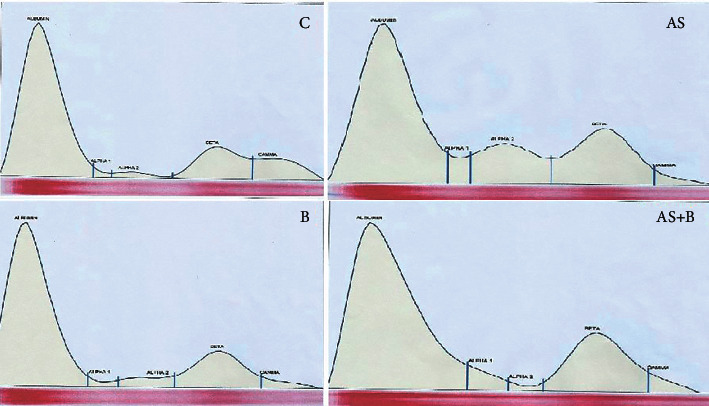
Electrophoretic changes of serum proteins in different groups.

The highest globulin levels were reported in the C and B groups (2.80 ± 0.05 and 2.31 ± 0.47, respectively), and the lowest levels were reported in the AS group (1.02 ± 0.31). The dissimilarity between the AS, AS + B, and control groups was significant. Albumin showed the highest values in the control group. The AS and AS + B groups were significantly different from the C group; moreover, there was a significant difference between the B and AS groups. The highest A/G ratio was observed in the AS group, and the lowest ratio was observed in the control group. Plasma total protein showed the highest value in the B group (6.5 ± 0.42) and the lowest value in the AS group (4.78 ± 0.38). In addition, alpha globulins showed higher values in the arsenic group than other groups (*P* = 0.032) (Table 4 and Figure 1).

**Figure 2:**
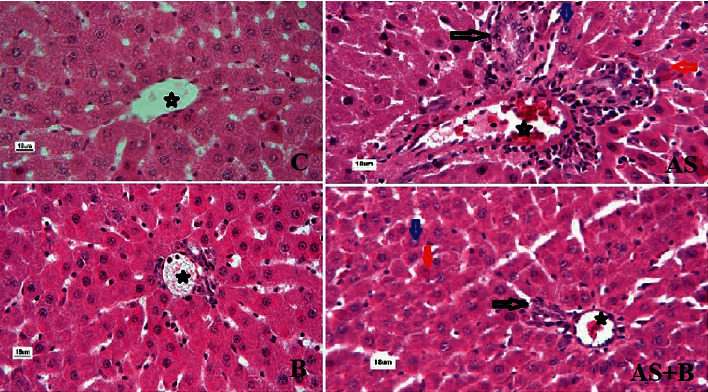
Histopathological changes of liver tissue in different groups. The star shows the central venous location, a black arrow pointing to the inflammatory cells, a blue arrow indicating to the cytoplasm vacuolation, and a red marker pointing the hepatocyte necrosis. magnification 400; H and E staining.

Pathological changes were compared between the different groups based on quantitative criteria. Infiltration of the inflammatory cells was highest in the AS group and lowest in the B group. The highest central portal venous hyperemia was in the AS group, with a mean of 3.74, while the lowest amount was in the control group. The highest cytoplasm vacuolation was seen in the AS group, and the lowest was in the B group. Moreover, vacuolation showed a significant decrease in the AS + B group compared to the AS group. With a mean of 2.67, hepatocyte necrosis was highest in the AS group and lowest in the B group. In the AS + B group, this reduction was not significantly different from the control group (Table 5 and Figure 2).

**Figure 3:**
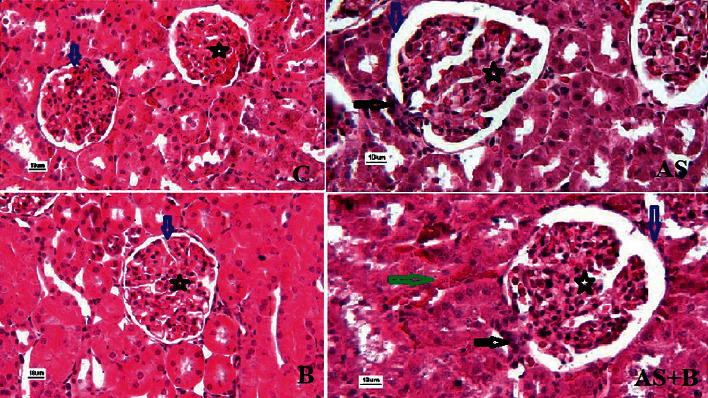
Histopathological assessments of renal tissue in different groups. Star is representing glomeruli, black arrow showing inflammatory cells, a blue arrow pointing the distance between Bowman's capsule membrane, and the green arrow showing hyperemia.

Table 6 shows all the pathological changes in the kidney tissue. There are four criteria, that is, the penetration of inflammatory cells, hyperemia, hemorrhage, and interstitial space in the Bowman capsule. The highest mean of infiltration of the inflammatory cells was in the AS group (3.69 ± 0.49), while the lowest was in the control group (0.03 ± 0.02). Vascular hyperemia has the greatest amount in the AS and AS + B groups, showing significant differences from the other groups. Moreover, the highest interstitial space in the Bowman capsule and its abnormal structure were in the AS group; however, this value was in a normal range in the control and B groups. The highest mean of bleeding was reported in the AS group (3.26), and the lowest was in the control group (0.03) (Figure 3).

## 4. Discussion

Due to the increased spread of agricultural and metallic pesticides in the environment, the amount of arsenic in the ecosystem is increasing. Pollution caused by these heavy metals has a chronic and long-term poisonous effect on wildlife [25]. Therefore, the use of chelator and antioxidant compounds is recommended to decrease the poisoning effects of heavy metals [26]. The present study confirmed that arsenic poisoning could cause changes in the hematological and biochemical parameters of the liver and kidneys, affecting the functions of these organs. On the other hand, liver damage can affect the total protein and electrophoretic pattern of other blood proteins. In addition, due to the oxidative function of arsenic in the body, we observed a decrease in enzymatic antioxidants and increase in oxidative damage to macromolecules. In this regard, the results indicate the positive performance of broccoli in controlling the hematological, biochemical, and enzymatic damage of arsenic.

The decreased levels of WBC and RBC were more severe in the AS group than in the other groups. Previous studies have shown that the concentration and route of administration of arsenic can decrease or increase the number of red blood cells [27].

Arsenic reduces the number of blood cells through inhibiting cell activity, antimycotic properties, stimulating oxidative stress, reducing cellular antioxidants, and increasing cell involvement in immune processes [28].

In other studies, the effect of arsenic on blood parameters has been implicated as a disorder of the Heme synthesis pathway, which is the beginning of the systemic effects of arsenic poisoning [29].

However, in a recent study, it was reported that doses of 300, 2000, and 4000 mg/kg of broccoli extract in 28 days showed no signs of poisoning by measuring the hematological, biochemical, and hepatic parameters [30]. Moreover, it is suggested that broccoli enhances the hematological parameters [31]. In another study, reduced production of RBC and WBC [32], oxidative stress in the liver, and alterations in the hematological parameters were reported to be caused by arsenic poisoning in fish [33]. Accordingly, the liver is one of the most important and active sites in the storage, redistribution, and detoxification of pollutants [34], and there are several tests to evaluate the liver. AST and ALT are used for checking the hepatocyte's damage, and they are also indicators of the liver's biosynthetic capacity. ALT is a particular indicator for hepatocyte necrosis. Not only did the AST increase in liver injury, but also it was involved in damage to other tissues, such as the heart, bones, skeletal muscles, and kidneys. AST and ALT levels increased when drugs and toxins caused liver necrosis [35]. Increased levels of AST and ALT have been reported in acute and chronic liver poisoning [35]. In parallel pathological studies, inflammation, necrosis, and apoptosis of the liver following arsenic poisoning in mice have confirmed liver damage. On the other hand, oxidative damage is one of the significant concerns in arsenic poisoning cases. Increasing the antioxidant potential of arsenic contaminants is one of the most important strategies to prevent damage to this toxic metal. In addition, the nuclear factor (erythroid-2 related), factor 2 (Nrf2), and the Nrf2-regulated signaling pathway are among the most important ways to control oxidative damage that can maintain cellular redox homeostasis against metal poisoning [36]. Hence, activation of the Nrf2 transcription gene can cause an upregulated number of antioxidant genes and control oxidative damage [37]. According to the action mechanism of arsenic [36], and in our case, broccoli, due to its special polyphenolic and strong antioxidant potential, may activate this pathway. This process may control oxidative damage to the liver and kidney and reduce ASL and ALT levels.

Since total protein, albumin, and globulin are produced by the liver, its damage can alter the synthesis of these compounds. Any condition causing hepatocellular damage reduces the level of albumin and globulin, like what happens in arsenic poisoning [38]. It is worth noting that the *α*_1_ fraction is mainly related to *α*_1_ antitrypsin. Arsenic-induced inflammation in liver damage causes an increase in *α*_1_ antitrypsin as an acute-phase reactant. Moreover, the *α*_2_ region contains *α*_2_ macroglobulin and haptoglobin [39]. The increase in *α*_2_ macroglobulin in the nephrotic syndrome, as well as the increase in haptoglobin in the inflammation of the liver, occurred as a result of arsenic poisoning [40].

Furthermore, the induction of oxidative stress and the binding of arsenic to protein groups containing Sh-thiol can reduce the synthesis and function of proteins in the body. The results showed that reducing oxidative stress using antioxidant-rich substances such as broccoli could increase the number of serum proteins [41].

The kidney damage induced by arsenic poisoning inhibits the antioxidant system functions and results in immunosuppression [42]. Moreover, arsenic poisoning of the kidney affects the regulation of proteins responsible for renal reabsorption and secretion, which causes elicited tubular damage [43, 44]. Elevated creatinine and urea concentration are the result of inflammatory and pathological damage in the glomerulus structures due to arsenic poisoning in the kidney. Because of the potential of quercetin in the detoxification of acetaminophen poisoning [45], concomitant administration of broccoli in reducing liver, kidney, and hematology arsenic poisoning can be helpful.

Although inorganic arsenic is more toxic than the organic one, oxidative damage mediated by ROS species is a common mechanism of poisoning in both substances. Moreover, cascading mechanisms of superoxide-free radical formation and decreased glutathione levels increase the susceptibility of cells to arsenic poisoning. During human and animal contact with arsenic, ROS/RNS production increases oxidative stress and causes damage to macromolecules, including lipids, which can increase MDA levels [46–48]. The overall result of this damage is different diseases of the liver, kidneys, nervous system, gastrointestinal tract, and reproductive system [49, 50]. Antioxidant substances such as vitamins C and E and quercetin, extracted from broccoli, increase the concentration of antioxidant enzymes responsible for preventing oxidative damage [46, 51]. Therefore, the results showed that in arsenic poisoning conditions, broccoli extract causes an increase in CAT, SOD, and GPx while decreasing MDA, thus reducing liver and kidney damage. Moreover, the results of the present study were in line with the research conducted by Sharma and Sangha, in which the multimechanistic protective effects (especially the antioxidant actions) of broccoli extract on Triazophos neurosplenic toxicity were shown [52].

## 5. Conclusion

Following the fact that the most dangerous effect of arsenic poisoning is increased oxidative stress, we showed a significant decrease in glutathione peroxidase and superoxide dismutase as well as an increase in MDA in the AS group. There was also an increase in the pathological damage to the liver and kidney tissue and the levels of AST, ALT, urea, and creatinine enzymes. On the other hand, due to the importance of the liver and kidneys in metabolism and excretion, their damage reduces the level of albumin and globulin. Our findings also showed reduced hemoglobin and red and white blood cells in the hematological parameters. Overall, we were able to reduce the antioxidant potentials of broccoli and prevent arsenic poisoning at the molecular level. Molecular studies on the action mechanisms of broccoli in alleviating the effects of arsenic poisoning are recommended to reveal the fundamental basics behind the results of the present study.

## Figures and Tables

**Table 1:** Changes in hematology parameters in the different groups of the study.

Parameters	Groups				
	Control	AS	B	AS + B	*P* value
WBC (per mm^3^)	7980.9 ± 950.3	7180.45 ± 322.6	1340 ± 230.64^a,b^	11548 ± 451.7^a,b^	0.034
RBC (×10^6^/*μ*L)	5.30 ± 0.81	4.37 ± 0.99^a^	6.03 ± 1.08^a,b^	5.56 ± 0.42^b,c^	0.049
HGB (g/dL)	14.88 ± 0.65	12.86 ± 1.43^a^	14.50 ± 1.10^b^	13.68 ± 1.00	0.043
PCV (%)	43.22 ± 3.76	41.62 ± 3.03	44.16 ± 1.57	41.56 ± 1.40	0.356
Neutrophils (%)	37.40 ± 1.67	42.60 ± 12.89	39.60 ± 6.18	39.80 ± 14.46	0.883
Lymphocytes (%)	58.80 ± .04	54.80 ± 12.35	55.00 ± 10.93	56.40 ± 15.91	0.942
Monocytes (%)	2.00 ± 0.00	1.60 ± 1.94	1.60 ± 0.54	1.00 ± 0.5	0.585
Eosinophils (%)	1.80 ± 0.44	0.84 ± 0.43	1.80 ± 0.83	1.60 ± .041	0.492

All values are presented as mean ± SEM. The letter (a) indicates a comparison with the control group, the letter (b) indicates a comparison with the AS group, and the letter (c) indicates a comparison with the B group. All data were analyzed using one-way ANOVA, and a post hoc test was performed by Tukey test (*n* = 8, *P* < 0.05).

**Table 2:** Evaluation changes in the biochemical parameters of the liver and kidney between groups.

Parameters	Groups				
	Control	AS	B	AS + B	*P* value
ALT (U/L)	28.4 ± 2.87	74.3 ± 1.90^a^	30.20 ± 4.25^b^	58.60 ± 6.60^a,b,c^	0.034
AST (U/L)	68.20 ± 4.80	142.25 ± 8.63^a^	62.60 ± 4.07	78.38 ± 12.38	0.049
ALP (U/L)	170.50 ± 12.24	245.82 ± 18.39^a^	183.22 ± 11.15^b^	190.34 ± 20.70^b^	0.043
Creatinine (mg/dl)	0.37 ± 0.12	1.42 ± 0.18^a^	0.52 ± 0.25^b^	0.97 ± 0.26^a,b^	0.036
Urea (mg/dl)	31.30 ± 8.50	50.18 ± 7.80^a^	32.10 ± 6.54 *α*,^b^	42.07 ± 11.03	0.048

All values are presented as mean ± SEM. The letter (a) indicates a comparison with the control group, the letter (b) indicates a comparison with the AS group, and the letter (c) indicates a comparison with the B group. All data were analyzed using one-way ANOVA, and a post hoc test was performed by Tukey test (*n* = 8, *P* < 0.05).

**Table 3:** Investigation of changes in oxidative stress biomarkers.

Parameters	Groups				
	Control	AS	B	AS + B	*P* value
CAT (nmol/ml)	7.31 ± 3.24	5.96 ± 0.88	9.38 ± 3.28	6.63 ± 1.69	0.197
GPx (nmol/ml)	5.12 ± 0.67	1.84 ± 0.97^a^	4.22 ± 2.04^a,b^	2.95 ± 1.6^a,b,c^	0.049
SOD (nmol/ml)	66.16 ± 7.49	23.22 ± 10.44^a^	64.11 ± 10.05^b^	32.45 ± 5.66^a,b,c^	≤0.01
MDA (nmol/ml)	4.73 ± 1.76	12.45 ± 1.20^a^	3.32 ± 1.96^b^	5.25 ± 2.64^b,c^	≤0.01
TAC (µmol/ml)	450.57 ± 37.58	220.29 ± 69.53^a^	858.47 ± 25.49^a,b^	628.40 ± 54.71^a,b,c^	≤0.01

All values are presented as Mean ± SEM. The letter (a) indicates a comparison with the control group, the letter (b) indicates a comparison with the AS group, and the letter (c) indicates a comparison with the B group. All data were analyzed using one-way ANOVA, and a post hoc test was performed by Tukey test (*n* = 8, *P* < 0.05).

**Table 4:** Serum protein values by electrophoresis in the studied animals.

Parameters	Groups				
	Control	AS	B	AS + B	*P* value
Globulin (g/dl)	2.80 ± 0.05	1.02 ± 0.31^a^	2.31 ± 0.47^b^	1.25 ± 0.36^a,c^	≤0.01
Albumin (g/dl)	4.81 ± 0.07	3.25 ± 0.51^a^	4.56 ± 0.36^b^	3.58 ± 0.37	≤0.01
A/G	1.71 ± 0.65	3.18 ± 0.49^a^	1.9 ± 0.28^b^	2.86 ± 0.89^a,b^	≤0.01
Total protein (g/dl)	6.25 ± 0.48	4.78 ± 0.38^a^	6.50 ± 0.42^b^	5.69 ± 0.37^a,c^	≤0.01
*α*-Globulin (g/dl)	0.50 ± 0.12	0.68 ± 0.35^a^	0.58 ± 0.04	0.52 ± 0.12	0.032
*β*-Globulin (g/dl)	0.62 ± 0.26	0.58 ± 0.14	0.70 ± 0.26	0.76 ± 0.04	0.215
*γ*-Globulin (g/dl)	0.84 ± 0.05	0.72 ± 0.03	0.70 ± 0.03	0.75 ± 0.02	0.453

All values are presented as Mean ± SEM. The letter (a) indicates a comparison with the control group, the letter (b) indicates a comparison with the AS group, and the letter (c) indicates a comparison with the B group. All data were analyzed using one-way ANOVA, and a post hoc test was performed by Tukey test (*n* = 8, *P* < 0.05).

**Table 5:** Evaluation of pathological changes in liver tissue in the experimental groups.

Parameters	Groups				
	Control	AS	B	AS + B	*P* value
Inflammatory infiltration	0.32 ± 0.01	4.5 ± 0.23^a^	0.03 ± 0.00^b^	2.38 ± 1.40^a,b,c^	0.047
Central venous congestion	0.06 ± 0.00	3.74 ± 0.67^a^	0.32 ± 0.05^b^	0.07 ± 0.6^b^	0.053
Cytoplasmic vacuolation	0.16 ± 0.09	2.22 ± 0.44^a^	0.11 ± 0.08^b^	1.12 ± 0.64^a,b,c^	≤0.01
Hepatocyte necrosis	0.73 ± 0.05	2.67 ± 1.32^a^	0.04 ± 0.03	1.02 ± 0.7^a,b,c^	0.034

All values are presented as Mean ± SEM. The letter (a) indicates a comparison with the control group, the letter (b) indicates a comparison with the AS group, and the letter (c) indicates a comparison with the B group. All data were analyzed using one-way ANOVA, and a post hoc test was performed by Tukey test (*n* = 8, *P* < 0.05).

**Table 6:** Evaluation of the histopathological structure of kidney tissue in the studied groups.

Parameters	Groups				
	Control	AS	B	AS + B	*P* value
Inflammatory infiltration cells	0.03 ± 0.02	3.69 ± 0.49^a^	0.04 ± 0.02^b^	2.38 ± 1.40^a,b,c^	0.037
Hyperemia	0.14 ± 0.01	2.12 ± 0.28^a^	0.17 ± 0.03^b^	2.04 ± 0.58^a,c^	0.051
Space of Bowman's capsule membrane	0.16 ± 0.09	3.10 ± 0.44^a^	0.28 ± 0.04^b^	2.95 ± 1.10^a,c^	0.01
Hemorrhage	0.03 ± 0.01	3.26 ± 0.98^a^	0.05 ± 0.04	1.48 ± 0.38^a,b,c^	0.027

All values are presented as Mean ± SEM. The letter (a) indicates a comparison with the control group, the letter (b) indicates a comparison with the AS group, and the letter (c) indicates a comparison with the B group. All data were analyzed using one-way ANOVA, and a post hoc test was performed by Tukey test (*n* = 8, *P* < 0.05).

## Data Availability

The datasets used and/or analyzed during the current study are available from the corresponding author on reasonable request.
